# Remote Sensing for Crops Spots Pests and Pathogens

**DOI:** 10.1021/acscentsci.3c00247

**Published:** 2023-03-07

**Authors:** Carolyn Wilke

In a vineyard in the Finger Lakes region of New York, a robot trundles along. Resembling a cable-covered WALL-E with a superlong neck, the rolling bot features sensors on top that scan a row of chardonnay grapevines, looking for signs of downy mildew.

Downy mildew, a fungal disease, splotches the undersides of grape leaves with yellow patches. The pathogen grows in its hosts’ cells and kills them—leaving brown, dead spots. Eventually it causes the plant to leak water and turn into a soggy mess. “Left unchecked in ideal environmental conditions, it would just effectively melt the plant,” says Katie M. Gold, a plant pathologist at Cornell University. A fast-spreading pathogen, downy mildew can explode across a vineyard in just a couple of days. A trained eye could catch an outbreak—if someone’s around.

By the time a person examines an afflicted vine, it might be too late. So Gold and her colleagues have built this robot to automatically detect the disease. The robot’s spectral sensors catch specific wavelengths of visible and infrared light bouncing off the leafy vines and make a diagnosis. “It’s just as good as an expert looking at the same picture,” Gold says.

**Figure:**
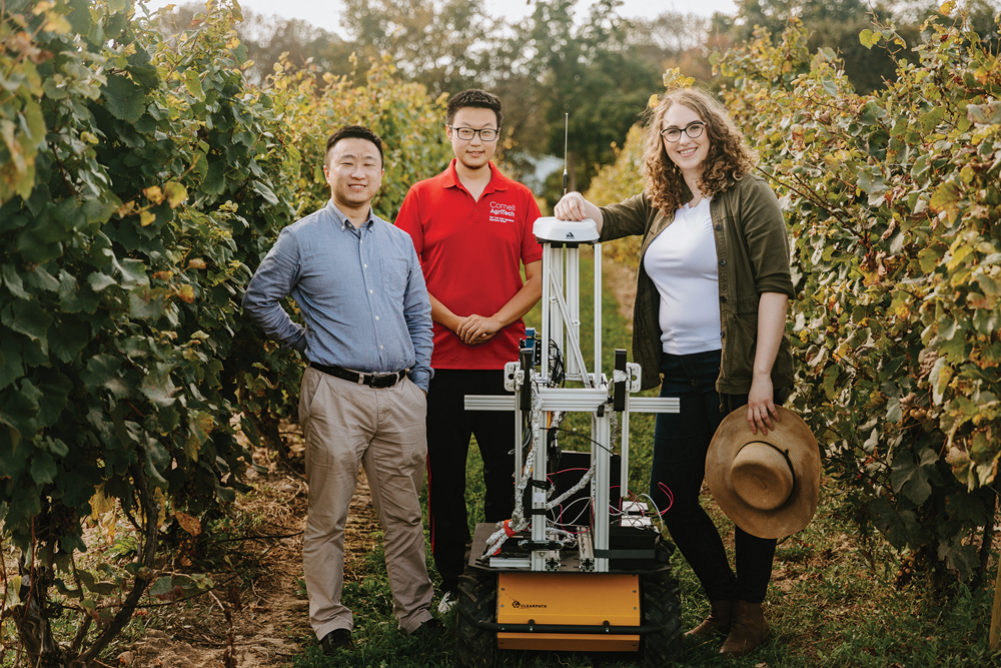
At Cornell University, Katie M. Gold (right) and colleagues Yu Jiang (left) and Leo Liu (center) are developing disease-spotting robots that could be automatically directed to an area of the vineyard according to what satellites detect from space. Credit: Allison Usavage

Meanwhile, small satellites orbiting high above the vineyard log the same spectral signals. They use a statistical approach to identify whether the plants in their pixels may be infected. The team is figuring out how to make the satellites and robots work together so that the satellites can identify a potential outbreak and its location in a vineyard at a very early stage of infection and then deploy the robot there to check it out.

Gold is one of several researchers seeking to snoop on the chemical communication of plants from afar using remote sensing. Their goal is to catch the hints plants give about their inner workings—subtle signs related to their pigments, defense chemicals, or structural compounds—and then clue in farmers earlier to pests and pathogens. In the case of downy mildew, if a grower can catch it before the infection finds its way across the field, they can avoid dousing their entire fields with fungicides and instead treat only the afflicted areas, potentially protecting against losses while helping decrease the amount of agrochemicals used. Scientists are applying the same reasoning to reduce the amount of fertilizer farmers apply to their crops, helping their bottom line and avoiding excess chemicals’ running off into the environment.

## Plants’ chemical communiqués

Unlike humans, whose immune systems learn as they face different threats, plants enter the world with all the immune defense systems they’ll ever have, Gold says. Their response to infections or pests is based on signaling, which turns on genes that produce defense compounds or that broadcast news of the attack throughout the plant. These chemical messages occur even at early stages of the disease, possibly before the infection can spread. That early messaging creates an opportunity to snoop on the plant through spectroscopy.

“We know that this behavior exists in plants and exists among all plants,” says Jordan A. Dowell, a plant biologist at the University of California, Davis. So researchers are building libraries of spectra collected from plants in different conditions. The goal is to link a plant’s chemical responses to, for example, fungal infection or attack by a leaf-munching bug. Scientists can do these sorts of studies in greenhouses with groups of plants or in fields using drones or cameras atop towers. In each case, researchers need to balance how detailed their view of plants is, how frequently they can collect data, and how many wavelengths they can gather and how closely those are spaced.

To detect downy mildew on grapes, Gold’s team is working with satellites carrying multispectral sensors, which sense light at just a few wavelengths. The signals they capture are more general stress indicators than ones specific to the disease, but the team is working on moving to hyperspectral imaging, a technique that collects spectral signals across a huge swath of wavelengths. Using different cameras, researchers can capture wavelengths in the range of 250–2,500 nm. That information is resolved into narrow bands—for instance, 10 nm wide, providing a trove of data on the plants. Then with statistical or machine learning approaches, researchers can connect those spectral bands to a plant’s condition and what’s occurring inside a plant.

“Hyperspectral remote sensing is sensitive to the chemical composition of plants,” says Philip A. Townsend, an ecologist who specializes in remote sensing at the University of Wisconsin–Madison. All those detected signals are based on the interaction of light with chemical bonds in the plant’s molecules. Some wavelengths correspond to plants’ proteins, starches, and lignin, for instance. Other features may require further investigation, such as a measurement of nutrients to learn their molecular basis. The level of detail offered by hyperspectral imaging can reveal distinct clues to what the plant is facing. And for some diseases, scientists have found ways to detect plants’ sicknesses before they’re visible to human eyes.

## Getting ahead of disease

In 2013, the pathogen *Xylella fastidiosa*, which infects a wide variety of plants, was detected for the first time in Europe. A known nuisance in North American vineyards and South American orange groves, the insect-borne bacterium cropped up in olive trees in southern Italy. As the pathogen spreads, it withered trees and scorched their leaves. “At early stages, it cannot be detected visually, as it takes months to develop,” says Pablo J. Zarco-Tejada, a remote-sensing scientist at the University of Melbourne. But during that time, up to the first year of the infection, disease can spread to other trees.

“At this moment, there is no cure,” Zarco-Tejada says. The only way to stop the bacteria from spreading is to destroy infected trees, he adds. A 2020 study estimated that the pathogen could cost European olive growers tens of billions of euros over the coming decades.

But Zarco-Tejada and his team have an eye in the sky: planes they fly over hectares of olive and almond trees, which *X. fastidiosa* has also infected, in Italy and Spain. Outfitted with thermal and hyperspectral cameras, the aircraft have imaged hundreds of thousands of trees.

**Figure:**
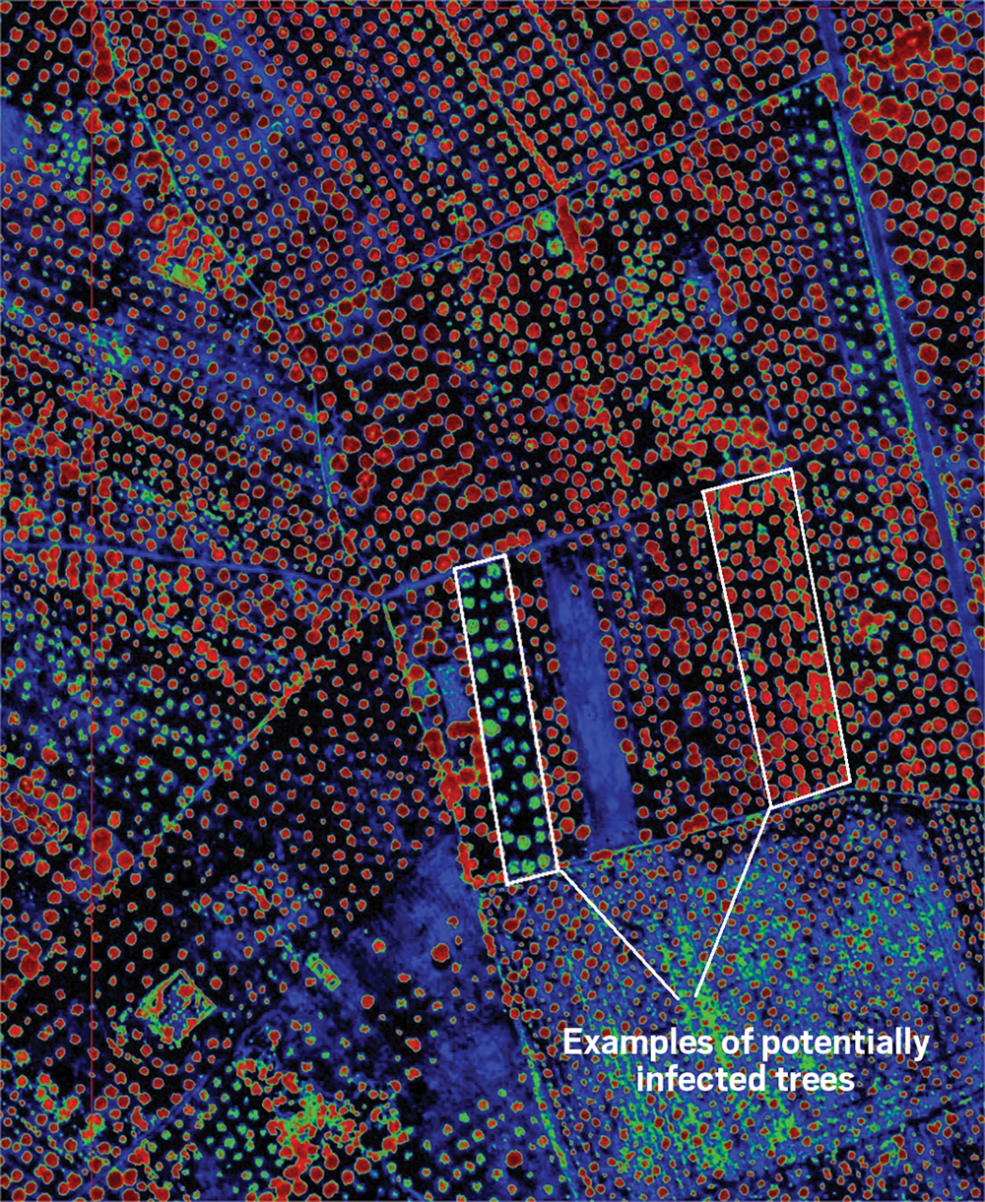
This aerial image of olive fields in southern Italy is based on blue, red, and near-infrared spectral windows taken by a hyperspectral camera. It captures information that can’t be detected with the naked eye—whether these trees are in the early stages of an infection with the pathogen *Xylella fastidiosa*. The trees’ crowns appear as dots. Potentially infected ones are lighter red and green. Credit: Pablo J. Zarco-Tejada

From all those data, the team extracted plant traits related to infection, such as changes to the transpiration and amounts of certain pigments, including chlorophylls and anthocyanins, and found signatures of the bacterial infection.

The researchers validated their method of spotting infections by comparing their results against DNA-sampling data that they collected confirming or denying the presence of microbes in the trees. The spectral signatures could be used to detect at least 80% of *X. fastidiosa*-infected trees, including those that were visually asymptomatic. “We could detect it with remote sensing before the plant pathologists could see it,” Zarco-Tejada says.

Because the bacteria make it look like the olive trees are experiencing drought stress, sometimes uninfected trees were misclassified, Zarco-Tejada says. So the team sought to untangle the symptoms of *X. fastidiosa* from those of other threats, including drought and a fungal infection. The researchers compared hyperspectral and thermal images of trees afflicted with these various conditions, and they were able to find more specific spectral fingerprints, achieving an accuracy of 92% in detecting the various plant stresses.

Zarco-Tejada’s team is now adding more species, such as peaches and citrus, to its repertoire. “During COVID, the whole world has become aware of how important the early detection of the infection is,” he says. This is true for plants too as new pathogens arise, current threats spread through international trade, and some from the past reemerge with a warming climate, he adds.

Researchers, including Zarco-Tejada and others, are also working to link what they see in the spectra to the processes inside plants. “We can learn something about the biology of the disease and how it progresses by looking at that chemical fingerprint,” Townsend says. Recently, scientists found that hyperspectral imaging could distinguish different sugar beet diseases on the basis of how each disease changed the plants’ levels of chlorophylls, carotenoids, flavonoids, and some phenols.

Researchers are starting to combine hyperspectral imaging with studies that screen for genetic variation in a large number of individuals. This combined approach could look for genetic differences that may be favorable for crops. Meanwhile, others are scouring hyperspectral data for signatures of other plant traits, such as flavor characteristics or disease resistance.

Hyperspectral imaging isn’t accessible to most farmers yet, Townsend says, and they may not need that level of sensing power anyway. As scientists sift through hyperspectral data to determine plants’ disease signatures, they can home in on some choice wavelengths that can be applied in far-cheaper multispectral sensors.

## Making plants shine

There’s another way to tell if plants are stressed: genetically engineer them so they reveal what’s ailing them.

InnerPlant, a start-up in Davis, California, is doing this by developing plants that glow in response to stressors such as pathogens and drought. Through RNA sequencing, scientists at the company have studied the gene activity of plants plagued by insect infestations, a lack of water, and insufficient phosphorus. Plants react to such threats rapidly and often in a very specific way, says Randy Shultz, a plant biotechnologist at the company. For instance, even though the visible symptoms may take days to develop, a soybean plant responds within 2 h of a certain fungal pathogen penetrating its tissue and starts turning on genes that are part of its defense, Shultz adds.

**Figure:**
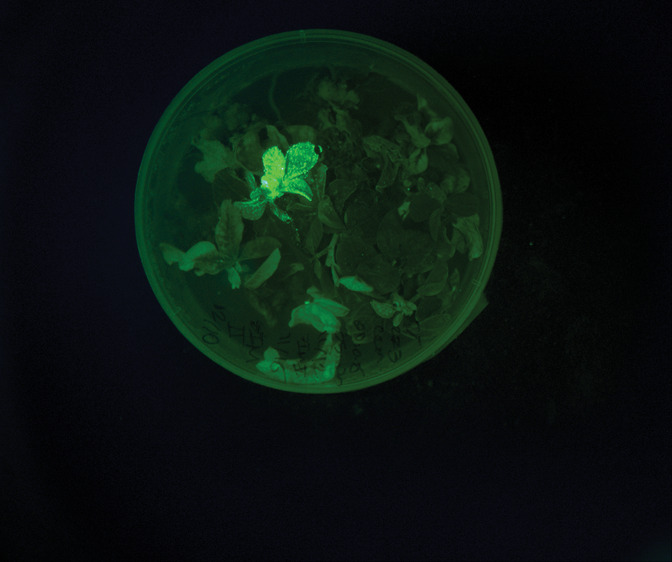
The start-up InnerPlant is creating plants, such as this soybean, that glow in response to drought, fungal infection, or other stressors. Credit: InnerPlant

To make the sensors as effective as possible, InnerPlant zeroed in on genes that were activated in a big way by specific attacks or nutritional deficiencies. The activity of some genes jumped by two or three orders of magnitude. Then the company’s scientists can splice in a gene for a fluorescent protein right next to the stress-activated gene so that the stressor will create a fluorescent signal. With several proteins that glow at different wavelengths, the company may eventually be able to build plants that can report multiple threats.

The researchers saw that a drought-sensing tomato plant started to boost its fluorescence some 24–28 h after watering stopped. Once the plant was watered again, the fluorescence returned to baseline levels.

For fluorescing plants to be useful to farmers, their glow needs to stand out from sunlight reflected off the leaves. So far, InnerPlant’s team has found that it can detect the fluorescence from plants in a sunlit field with ground-based detectors. The company’s goal is to trigger fluorescent responses that could be spotted from space by a satellite. The company is growing prototypes of soybean plants engineered to glow when enduring an attack from a fungal pathogen, and the company plans to test them in the field this year.

InnerPlant is working on equipping tractors with fluorescence detectors through a partnership with John Deere, Shultz says. A satellite could detect hectare-level signals of stress and steer a tractor to the afflicted crop. That tractor could then get even more granular data. InnerPlant has worked with Deere to model how earlier warning about pathogens or pests could affect pesticides applied to the field. The companies’ analysis suggests that turning crops grown over large areas, such as cotton and soybeans, into glowing stress sensors could reduce pesticide application by 75%. This change could save a chunk of the tens of billions of dollars that analysts estimate that the world’s farmers spend on pesticides each year.

It may be some time before these innovations can be fine-tuned and tested so that farmers everywhere have access to glowing plants or the ability to see their fields with hyperspectral imaging. That said, efforts announced by NASA and the European Space Agency to put hyperspectral equipment into orbit by 2030 are promising signs of what is to come. Plant scientists will have their work cut out for them on the ground, developing methods to deal with the deluge of data to better understand what plants are trying to tell them.

*Carolyn Wilke is a freelance contributor to**Chemical & Engineering News**, the independent news outlet of the American Chemical Society*.

